# Novel insights into the dependence of adsorption-desorption kinetics on particle geometry in chiral chromatography

**DOI:** 10.1007/s00216-024-05186-z

**Published:** 2024-02-15

**Authors:** Chiara De Luca, Greta Compagnin, Chiara Nosengo, Giulia Mazzoccanti, Francesco Gasparrini, Alberto Cavazzini, Martina Catani, Simona Felletti

**Affiliations:** 1 Department of Chemical, Pharmaceutical and Agricultural Sciences, University of Ferrara, via L. Borsari 46, Ferrara, 44121 Italy; 2 Department of Drug Chemistry and Technology, “Sapienza” Università di Roma, P.le A. Moro 5, Rome, 00185 Italy; 3 Council for Agricultural Research and Economics, CREA, via della Navicella 2/4, Rome, 00184 Italy; 4 Department of Environmental and Prevention Sciences, University of Ferrara, via L. Borsari 46, Ferrara, 44121 Italy

**Keywords:** Superficially porous particles, Adsorption-desorption kinetics, Sub 2-$\mu$m fully porous particles, Chiral chromatography

## Abstract

**Abstract:**

The existence of slow adsorption-desorption kinetics in chiral liquid chromatography is common knowledge. This may significantly contribute to worsening the efficiency and kinetic performance of a chromatographic run, especially when high flow rates are employed. Many attempts and protocols have been proposed to access this term, the so-called $c_{ads}$, but they are based on different (theoretical) assumptions. As a consequence, no official method is available for the estimation of the adsorption-desorption kinetics term. In this work, a novel approach to access $c_{ads}$ is presented. This procedure combines experimental results obtained with kinetic and thermodynamic measurements. The investigations have been performed on two zwitterionic teicoplanin chiral stationary phases (CSPs) based on 1.9 $\mu$m fully porous and 2.0 $\mu$m superficially porous particles (FPPs and SPPs), using Z-D,L-Methionine as probe molecule. Kinetic studies have been performed through the combination of both stop-flow and dynamic measurements, while adsorption isotherms have been calculated through Inverse Method. This study has confirmed that, on both particle formats, analyte diffusion on the surface of the particle is negligible, meaning that adsorption is localized, and it has been demonstrated that adsorption-desorption kinetics is strongly dependent on particle geometry and, in particular, on the loading of chiral selector. These findings are fundamental not only to unravel novel aspects of the complex enantiorecognition mechanism but also to optimize the employment of CSPs for ultra-fast and preparative applications.

**Graphical abstract:**

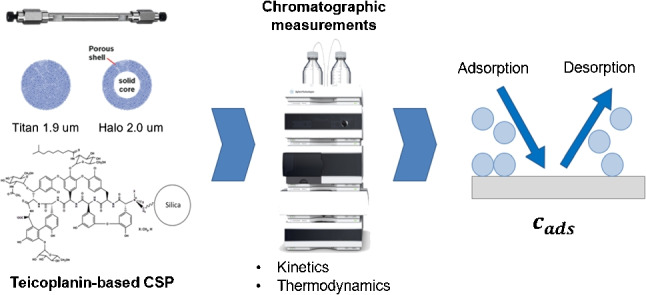

## Introduction

In the last decades, particular attention has been paid on the development on more and more efficient packing particles. Indeed, either fully (FPPs) or superficially porous particles (SPPs) with diameter less than 2 $\mu$m have been developed. Nevertheless, this innovation was delayed for chiral stationary phases (CSPs) due to a series of challenges, especially in the functionalization and packing procedures, but also related to a lack of fundamental information about mass transfer and chiral recognition mechanism [1, 2]. As a matter of fact, conversely to common expectations, it has been experimentally observed that in chiral chromatography (for defined conditions) SPPs do not always outperform FPPs in terms of kinetic performance [3–7]. This could be attributable to the existence of slow adsorption-desorption kinetics between enantiomers and the chiral selector. Indeed, the diastereomeric transient complexes between the chiral selector and enantiomers are only established if a specific spatial orientation of molecules is achieved. As a consequence, in some cases, enantiorecognition mechanism can take a significant amount of time, which contributes to increase the peak broadening, even at small flow rates and for small molecules [8–13].

Over the years, different attempts and protocols have been proposed to access this term, but they are based on different (theoretical) assumptions and approximations [6, 9, 11]. As a consequence, no official method is available for the estimation of the adsorption-desorption kinetics term.

Even if the complete understanding of what happens at a molecular level has not been accessed yet, some clues helpful to explain this behavior can be found in the intrinsic differences between particles (e.g., selector loading and pore accessibility) coupled to important findings about molecular diffusion occurring on the surface of the chiral particle [7, 10].

To further shed light on this very complex topic, all aspects, kinetic characteristics and thermodynamic properties of both SPPs and FPPs need to be carefully taken into account and investigated. In this context, two zwitterionic teicoplanin-based CSPs prepared on 2.0 $\mu$m Halo SPPs (SPP-2.0) and on 1.9 $\mu$m Titan FPPs (FPP-1.9) have been considered and compared. Teicoplanin-based CSPs used in this work were obtained by covalently bonding teicoplanin to the bare silica by Gasparrini group, as reported in Ref. [4, 5].

Focus of this study is thus to highlight the intrinsic kinetic and thermodynamic differences between superficially and fully porous particle formats, functionalized with the same bonding protocol, with particular emphasis on how molecular interactions on adsorption sites can be influenced by particle geometry and how the density of chiral selector could affect adsorption-desorption kinetics.

In this work, for the first time, a novel approach based on previously obtained thermodynamic results has been applied for the calculation of the adsorption-desorption kinetics term. The great influence of particle geometry on band broadening has been experimentally demonstrated and possible suggestions for the improvement of kinetic performance of packing particles are presented.

## Theory

### Mass transfer kinetics

Kinetic performance of particles of different geometry is commonly evaluated and compared through the van Deemter equation reported in Eq. 1 in reduced coordinates [8]:

1 $$\begin{aligned} h= a(\nu )+\dfrac{b}{\nu }+(c_s+c_{ads})\nu \end{aligned}$$

with *h* the reduced plate height (=$H/d_p$, being *H* the plate height and $d_p$ the particle diameter), $\nu$ the reduced interstitial velocity, $a(\nu )$ the eddy dispersion, *b* the longitudinal diffusion, and $c_s$ and $c_{ads}$ the solid-liquid mass transfer and the adsorption-desorption kinetics terms, respectively. $\nu$ is defined as:

2 $$\begin{aligned} \nu =\frac{u_e\,d_p}{D_m} \end{aligned}$$

with $D_m$ the bulk molecular diffusion coefficient and $u_e$ (= $F_v/\pi r_c^2 \epsilon _e$, being $F_v$ the flow rate, $r_c$ the column radius and $\epsilon _e$ the external column porosity) is the interstitial velocity, i.e., the velocity of the mobile phase moving between particles [14]. The use of stop-flow measurements, like peak parking (PP), permits to evaluate both effective and molecular diffusion coefficients ($D_{eff}$ and $D_m$) [16–20]. Once these coefficients are known, the longitudinal diffusion term of Eq. 1, *b*, can be easily calculated for the first and the second eluted enantiomer (*i* = 1,2):

3 $$\begin{aligned} b_i=2(1+k_{1,i})\frac{D_{eff,i}}{D_m} \end{aligned}$$

with $k_{1,i}$ the zone retention factor (i.e., the retention factor referred to the interstitial volume [20]) of the *i*-th enantiomer, defined as:

4 $$\begin{aligned} k_{1,i}=\frac{t_{R,i}-t_e}{t_e} \end{aligned}$$

with $t_R$ the retention time and $t_e$ the time spent by a molecule in the interstitial volume. $k_1$ is correlated to the phase retention factor, *k*, through:

5 $$\begin{aligned} k_i=\frac{t_{R,i}-t_0}{t_0}=\frac{(1+k_{1,i})\epsilon _e}{\epsilon _t}-1 \end{aligned}$$

being $\epsilon _t$ the total porosity of the column.

$D_{eff}$ can be interpreted in the light of a diffusion model and in this work the time-averaged (or Knox) model has been employed, for which contributions inside and outside particles are considered to be additive [8, 15, 20]:

6 $$\begin{aligned} D_{eff}=\frac{\gamma _e D_m+\frac{1-\epsilon _e}{\epsilon _e}\big (1-\rho ^3\big )D_p}{1+k_1} \end{aligned}$$

where $\rho$ is the ratio between the radius of the core and that of the whole particle ($\rho$ is thus 0 for FPPs and 1 for non-porous ones). This equation permits the calculation of $D_{p,i}$, that is the intraparticle diffusivity. By applying the Knox model inside the particles, it can be defined as [8, 20, 31]:

7 $$\begin{aligned} D_{p,i}=\epsilon _p\gamma _p F(\lambda _m)D_m+ (1-\epsilon _p)K_i D_{s,i} \end{aligned}$$

where $\epsilon _p$ is the particle porosity (i.e., the fraction of volume occupied by pores), $\gamma _p$ a geometrical parameter called internal obstruction factor (taking into account the tortuosity of the mesopore pathways, their constriction and connectivity), $F(\lambda _m$) the hindrance diffusion factor (which accounts for the confinement of the analyte within narrow pores), $K_i$ the Henry’s constant of adsorption and $D_{s,i}$ the surface diffusion.

The solid-liquid mass transfer resistance term, $c_s$, is defined as [8, 46]:

8 $$\begin{aligned} c_{s,i} =\dfrac{1}{30}\dfrac{\epsilon _e}{1-\epsilon _e}\bigg [\dfrac{k_{1,i}}{1+k_{1,i}}\bigg ]^2\dfrac{1+2\rho +3\rho ^2-\rho ^3-5\rho ^4}{(1+\rho +\rho ^2)^2}\dfrac{D_m}{D_{p,i}} \end{aligned}$$

It is well known from achiral RP chromatography [21–26] that the major differences between core-shell and fully porous particles are diminished $a(\nu )$, *b*, and $c_s$ terms, due to the presence of the impenetrable inner core that reduces the physical space available for diffusion. Nevertheless, in case of chiral separations the contribution to band broadening by the adsorption-desorption kinetics cannot be neglected ($c_{ads}$ $\ne$0) [8, 9, 13]. This is true especially for the most retained enantiomer, since chiral recognition mechanism occurring between enantiomer and selector may take a significant amount of time, leading to an important band broadening due to slow adsorption-desorption kinetics. $c_{ads}$ is defined as:

9 $$\begin{aligned} c_{ads} =2\,\dfrac{\epsilon _e}{1-\epsilon _e}\dfrac{1}{1-\epsilon _p}\dfrac{1}{1-\rho ^3}\bigg (\dfrac{k_{1,i}}{1+k_{1,i}}\bigg )^2\bigg (\dfrac{k_p}{1+k_p}\bigg )^2\dfrac{D_m}{k_{ads}d_p^2} \end{aligned}$$

with $k_{ads}$ the kinetic adsorption constant and $k_p=(1-\epsilon _p)K_a/\epsilon _p$.

The quantification of $c_{ads}$ is very challenging, since it can be obtained through the subtraction of all the contribution to band broadening to *h* Eq. 1, once $a(\nu )$, *b* and $c_s$ have been calculated, or through Eq. (9) once $k_{ads}$ has been evaluated. On the one hand, $a(\nu )$ can be estimated both through theoretical measurements of trans-channel, short-range, inter-channel and trans-column eddy dispersions [8, 13] or by employing achiral compounds of similar retention with respect to the chiral probes under investigation. On the other hand, $k_{ads}$ can be determined through the microscopic model of chromatography, such as stochastic theory of chromatography [27–30]. However, all the approaches reported present some limitations, which make the accurate measurement of the adsorption-desorption kinetics difficult and challenging.

Nevertheless, some important information may come from thermodynamic measurements, since it is strictly correlated to adsorption-desorption kinetics [31], and the physical and geometrical characteristics of the particles. Indeed, it has been recently proven [6] that higher specific bonding density of chiral selector is directly related to larger binding constants responsible for slower adsorption-desorption kinetics (i.e., larger $c_{ads}$).

### Thermodynamics

#### Equilibrium-dispersive model

The simplest model of chromatography that takes into account both the contributions of the axial dispersion (molecular and eddy diffusions) and of the mass transfer kinetics is the so-called Equilibrium Dispersive (ED) model [31]. This model assumes an instantaneous equilibrium between the mobile (MP) and the stationary phase (SP) and all contributions related to nonequilibrium effects are lumped into an apparent axial dispersion term, $D_a$. In the case of enantiomeric separations, two partial differential mass balance equations are required for each analyte:

10 $$\begin{aligned} \frac{\partial C_i}{\partial t}+F\frac{\partial q_i}{\partial t}+u\frac{\partial C_i}{\partial z}=D_{a,i}\frac{\partial ^2 C_i}{\partial z^2} \hspace{1cm} \text {with}\quad i=1,2 \end{aligned}$$

with *u* the mobile phase linear velocity, *F* the phase ratio (=$V_S/V_m$ = (1-$\epsilon _t$)/$\epsilon _t$), $q_i$ and $C_i$ the concentrations of the *i*-th analyte adsorbed on the stationary phase and that in the bulk mobile phase, respectively. $q_i$ and $C_i$ are connected through a competitive isotherm equation, $q_i=f(C_1,C_2)$.

#### Inverse method and Bilangmuir isotherm model

Isotherm parameters have been calculated with the so-called Inverse Method through the iterative resolution of the system of mass balance equations (Eq. 10). Once an isotherm model has been chosen, theoretical band profiles are calculated and compared to experimental overloaded peaks previously measured. Then, a numerical optimization of the isotherm parameters is performed through the super-modified simplex method by minimizing the sum of the least squares between experimental and calculated profiles [32–34].

In this work, the competitive Bilangmuir isotherm model has been selected for the calculations of isotherm parameters. This type of isotherm accounts for a bimodal energy distribution, given by two different adsorption sites: one selective (responsible for diastereomeric or enantioselective interactions) and one nonselective (where both enantiomers behave identically) [31, 32]. For the reasons presented above it can be successfully applied to chiral separations [7, 10, 32, 34]. For two competitive components, it is written as:

11 $$\begin{aligned} q_i = \frac{q_{sel}^sb_{i,sel}C_i}{1+b_{1,sel}C_1+b_{2,sel}C_2}+ \frac{q_{nsel}^sb_{nsel}C_i}{1+b_{nsel}(C_1+C_2)} \hspace{2cm} i = 1,\,2 \end{aligned}$$

with $q^s$ the saturation capacity (equal for both enantiomers) and $b_i$ the adsorption equilibrium (binding) constant. Subscripts *sel* and *nsel* refer to selective and nonselective sites, respectively. It is worth noting that the absence of chiral interactions in nonselective sites leads to the same thermodynamic behavior of the two enantiomers, as suggested by the single value for the binding constant, $b_{nsel}$ (equal for the two species). The product $q^s\,b_i=a_i$ is called Henry’s constant of adsorption which defines the initial slope of the adsorption isotherm. Retention factors, $k_i$, are directly connected to $a_i$ through the following equation:

12 $$\begin{aligned} k_{i}=\big (a_{i,sel}+a_{i,nsel}\big )F \end{aligned}$$

It is worth mentioning that saturation capacities calculated with Inverse Method are referred to the stationary phase volume, $V_s$, through:

13 $$\begin{aligned} q^s=\frac{n_s}{V_s}=\frac{n_s}{(1-\epsilon _t)V_{col}} \end{aligned}$$

with $n_s$ the moles of analyte adsorbed on the stationary phase and $V_{col}$ the geometrical volume of the column. Equation 13 is valid for totally porous particles. Conversely, in case of core-shell particles a correction factor to account for the presence of the solid core, $V_{core}$, must be added to $q^s$, since the volume accessible to the analyte is only the porous shell volume, $V_{shell}$, defined as [34]:

14 $$\begin{aligned} V_{shell}&=V_s-V_{core}\nonumber \\&= (1-\epsilon _t)V_{col}-\rho ^3(1-\epsilon _e)V_{col}\nonumber \\&= V_{col}\big [(1-\epsilon _t)-\rho ^3(1-\epsilon _e)\big ] \end{aligned}$$

Hence, the saturation capacity for SPPs is expressed as:

15 $$\begin{aligned} q_{SPP}^s&=\frac{n_s}{V_{shell}}\nonumber \\&= \frac{n_s}{V_{col}\big [(1-\epsilon _t)-\rho ^3(1-\epsilon _e)\big ]}\nonumber \\&= \frac{q_s (1-\epsilon _t)}{(1-\epsilon _t)-\rho ^3(1-\epsilon _e)} \end{aligned}$$

## Experimental section

### Columns and materials

All solvents, reagents and the analyte (Z-D,L-methionine) were purchased from Merck Sigma-Aldrich (St. Louis, MO, USA). Titan monodispersed silica (1.9 $\mu$m, 120 Å, 282 m^2^/g), Halo silica (2.0 $\mu$m, 90Å, 125 m^2^/g, $\rho$=0.6) and teicoplanin chiral selector were from Merck Sigma-Aldrich (St. Louis, MO, USA). 100$\times$4.6 mm empty stainless steel columns were from IsoBar Systems by Idex (Erlangen, Germany). A 33 $\times$4.6 mm Micra column (Eprogen, Inc., USA) packed with 1.5 $\mu$m non-porous silica particles was purchased from DBA Italia s.r.l. (Italy) and employed for the estimation of bulk molecular diffusion coefficients. The mobile phase employed was ACN/H_2_O 85:15% (v/v) + 20 mM ammonium formate.

### Equipment

An UltiMate 3000 RS UHPLC chromatographic system from Thermo Fisher Dionex was used for the determination of van Deemter curves. This instrument consists of a dual gradient RS pump (maximum flow rate = 8.0 mL/min; pressure limit 800 bar under normal phase conditions), an in-line split loop well plate sampler, a thermostated RS column ventilated compartment and a diode array detector (UV Vanquish) with a low dispersion 2.5 $\mu$L flow cell. Detection wavelength was 214 nm. Two 350$\times$0.10 mm I.D. Viper capillaries (internal volume = 2.7 $\mu$L) were used to connect the injector to the column and the column to the detector. The extra-column peak variance at a flow rate of 1.0 mL/min was 4.0 $\mu$L^2^.

van Deemter curves were measured at 35^∘^C. Flow rates were changed from 0.2 mL/min up to 4.0 mL/min and injection volume was 1 $\mu$L. Peak parking and adsorption isotherm experiments were carried out on an Agilent 1100 Series Capillary LC system equipped with a binary pump system (maximum flow rate = 2.5 mL/min), an autosampler, a column thermostat and a photodiode array detector.

Adsorption isotherms were measured at a flow rate of 0.4 mL/min, injected volume of the racemic mixture was 5 $\mu$L and injected concentrations were as follows: 6, 12 and 25 g/L. Temperature was set to 35^∘^C.

**Table 1:** Geometrical and physico-chemical characteristics of Teicoplanin-based chiral particles and columns

Brand	$d_p$	A_s_	Pore size	Bonding density		$\epsilon _t$	$\epsilon _e$	$\epsilon _p$
/Particle type	$\mu$m	m^2^/g	Å	$\mu$mol/g	$\mu$mol/m^2^			
Titan	1.9	282	120	76	0.3	0.66	0.42	0.41
/FPP								
Halo	2.0	125	90	56	0.5	0.54	0.41	0.27
/SPP								

Brand: commercial silica name; particle type: FPP = fully porous, SPP = superficially porous; d_p_: particle diameter; A_s_: specific surface area; $\epsilon _t$: total porosity; $\epsilon _e$: external porosity; $\epsilon _p$: particle porous zone porosity

### Peak parking experiments

Effective, $D_{eff}$, and molecular, $D_m$, diffusion coefficients were estimated with peak parking experiments [16–20]. $D_{eff}$ is calculated through the following equation:

16 $$\begin{aligned} D_{eff}=\frac{\Delta \sigma ^2_{x,i}}{2\Delta t_p} \end{aligned}$$

with $\sigma ^2_{x,i}$ the spatial peak variance and $t_p$ the parking time. $\sigma ^2_{x,i}$ can be obtained using the following equation:

17 $$\begin{aligned} \sigma ^2_{x,i}=\frac{L^2}{N_i} \end{aligned}$$

with *L* the column length and *N* the number of theoretical plates returned by the software (calculated through the method of moments). Parking times were 0, 2, 5, 10, 20 and 30 min. Injected volume was 1 $\mu$L and the flow rate was 0.2 mL/min.

$D_m$ is estimated by performing peak parking in a column packed with non-porous particles (Micra column). In this case $D_m=D_{eff}/\gamma _e$, with $\gamma _e$ the external obstruction factor. This parameter can be measured through peak parking using thiourea, a molecule whose $D_m$ is known from literature ($D_m$= 1.33 $\times$ 10^-5^ [45]).

**Table 2:** Zone retention factor ($k_1$), selectivity ($\alpha$), effective diffusion coefficient ($D_{eff}$), reduced longitudinal diffusion coefficient (*b*), reduced solid-liquid mass transfer resistance coefficient ($c_s$), adsorption-desorption kinetics term ($c_{ads}$) measured on FPP-1.9 and SPP-2.0 Teicoplanin-based chiral particles

Column	$k_1$		$\alpha$	$D_{eff}\times 10^{-6}$		*b*		$c_s$		$c_{ads}$
	1^st^	2^nd^		1^st^	2^nd^	1^st^	2^nd^	1^st^	2^nd^	2^nd^
FPP-1.9	3.1	4.7	1.6	2.9	2.2	2.0	2.0	0.060	0.064	0.115
SPP-2.0	2.3	3.8	1.8	3.2	2.2	1.7	1.7	0.039	0.045	0.213

**Table 3:** Bilangmuir isotherm parameters calculated through Inverse Method for FPP-1.9 and SPP-2.0 columns

Column	Selective site			Nonselective site	
	$q^s_{sel}$	$b_{1,sel}$	$b_{2,sel}$	$q^s_{nsel}$	$b_{nsel}$
	(g/L)	(L/g)	(L/g)	(g/L)	(L/g)
FPP-1.9	0.99±0.10	0.038±0.001	1.32±0.04	125±3	0.022±0.002
SPP-2.0	0.67±0.05	0.055±0.001	1.34± 0.03	77±2	0.027±0.001

## Results and discussion

Table 1 lists the physico-chemical characteristics of particles and columns employed in this work [4, 5]. Information on particle diameter, specific surface area and pore size comes from manufacturers. Particles total porosity ($\epsilon _t$), external porosity ($\epsilon _e$) and particle porosity ($\epsilon _p$) have been experimentally determined through inverse size exclusion chromatography (ISEC) [5, 35, 36]. Specific bonding density ($\mu$mol/m^2^) has been determined through elemental analysis (see Ref. [5]) and it was found to be larger on SPPs with respect to FPPs, with a difference of 40%, despite that the functionalization protocol was performed under identical experimental conditions. This last finding has been observed also by other authors and with different chiral selectors [21, 34, 37]. The most likely hypothesis to explain this result is the different accessibility of intraparticle space during particle functionalization [34].

**Fig. 1:**
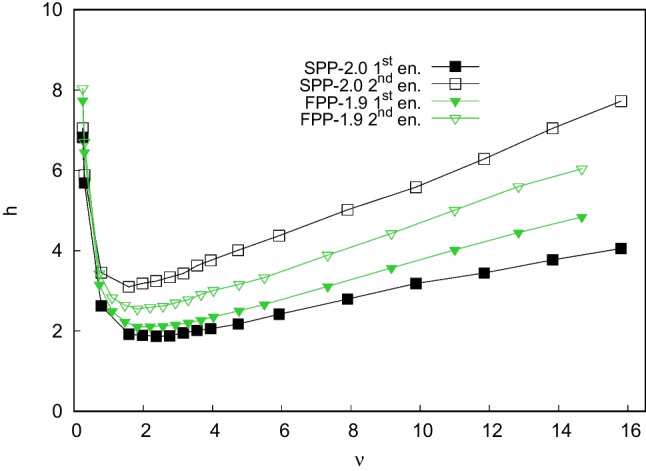
Reduced van Deemter curves of first enantiomer (full symbols) and second enantiomer (empty symbols) of Z-Methionine on FPP-1.9 column (green triangles) and SPP-2.0 column (black squares). *h*: reduced plate height, $\nu$: reduced interstitial velocity

### Kinetic measurements

In the first part of the study, experimental measurements and theoretical kinetic calculations have been performed for racemic Z-D,L-Methionine (Z-Met), a molecule bearing a carboxylic function, which represents the key fragment involved in the association process with the teicoplanin selector, on SPP-2.0 and FPP-1.9 zwitterionic teicoplanin-based columns. For the sake of completeness, the interested reader is referred to Ref. [5] for analytical chromatograms.

In order to evaluate the kinetic performance of the two columns, van Deemter plots have been measured and are shown in Fig. 1. From these plots, it can be seen that the curve of the first enantiomer measured on the SPP column lays below the curves measured on the FPP column, which reflects the expected trend. Conversely, the curve of the second eluted enantiomer on the SPP column is the less efficient if compared to its FPP counterpart. Indeed, the gap between van Deemter curves measured at $\nu _{max}$ ($\sim$15) for Z-Met enantiomers is considerably larger for SPP column (=4.2) in comparison to FPP (=1.2). This trend has already been observed with other chiral selectors [34].

At very small reduced velocity, $\nu$, where the longitudinal diffusion is the principal source of band broadening, the curves of the two enantiomers are almost perfectly superimposable. On the contrary, at higher $\nu$, more significant differences between values measured for the less and more retained enantiomers have been observed depending on the particle geometry.

In order to give an explanation to this behavior, all sources of band broadening have been investigated. Results of PP experiments are reported in Table 2. As it can be easily noticed, enantiomers of Z-Met are characterized by the same *b* on each column. The same observations reported in [6] can then be made. Indeed, in Ref. [6], it has been experimentally demonstrated that, by considering the Knox model, if $b_1=b_2$ then $D_{s,1}=D_{s,2}=0$ (with $D_s$ the surface diffusion coefficient). This indicates that the contribution to the intraparticle diffusivity of surface diffusion can be considered negligible, suggesting the localized nature of the adsorption process. This result is somehow expected since, from excess isotherm measurements, it has been demonstrated that a low-mobility water-rich layer covers the adsorbent surface hindering the mobility of the analyte molecules [7]. Little contributions of surface diffusion have been observed also in achiral HILIC mode [38], in chiral RP [13], chiral polar organic mode (POM) [10] and NP [6] chromatography.

As expected, *b* and $c_s$ values are smaller on SPPs than on FPPs, due to the minor particle porosity and the minor accessibility of intraparticle space [13, 23, 24]. However, since the contributions of longitudinal diffusion and the solid-liquid mass transfer resistance have similar effects on the overall efficiency (see Table 3) of both particle geometries, other kinetic phenomena may be at the origin of the variations observed in Fig. 1. To shed light on the substantial differences observed between the kinetic behavior of enantiomers on the two columns, a deeper investigation of the influence of other band broadening effects, namely eddy dispersion and adsorption-desorption kinetics, is pivotal.

The extent of efficiency gaps (see Fig. 1) may be an indicator of the contribution of the slower and stereochemical dependent selector-selectand association and adsorption process, directly influencing $c_{ads}$-term. Indeed, molecular modeling and X-ray investigations have demonstrated that the association and interaction mechanism between teicoplanin selector and analytes bearing a carboxylate fragment involves the formation of at least three hydrogen bonds [39–43]. Moreover, if the NH group is present in the ligand (as for Methionine) an additional fourth H-bond can be established between analyte and selector. It is obvious that the strength of this new interaction depends on the stereochemistry of the ligand, having a deep influence on the stabilization of the complex analyte-selector. As a matter of fact, the two enantiomers may behave and interact differently with the stationary phase.

Nevertheless, the estimation of the $c_{ads}$-term Eq. (1) can be very challenging and difficult. Even the most advanced approaches to the study of mass transfer in LC show that $c_{ads}$ cannot be independently evaluated. Indeed, the application of the so-called subtraction method to Eq. 1 leads to the sum of $a(\nu )$ and $c_{ads}$:

18 $$\begin{aligned} a_i(\nu )+c_{ads,i}\nu =h_i-\dfrac{b}{\nu }-c_{s,i}\nu \end{aligned}$$

**Fig. 2:**
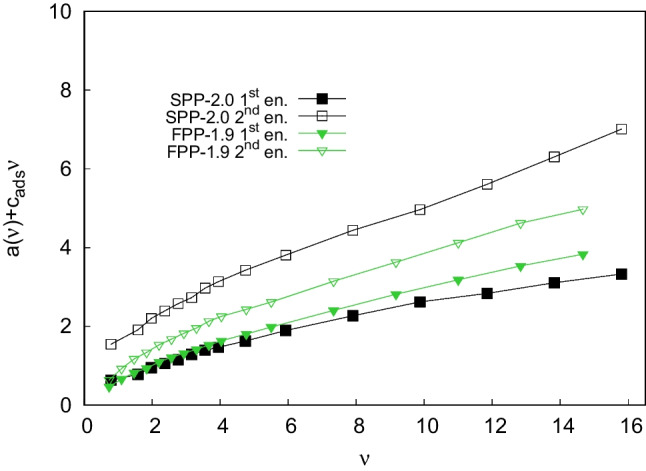
Eddy dispersion and adsorption-desorption kinetics ($a(\nu )+c_{ads}\nu$) contributions of first (full symbols) and second (empty symbols) eluted Z-Methionine enantiomers on FPP-1.9 column (green triangles) and SPP-2.0 column (black squares) as a function of the reduced interstitial velocity, $\nu$

In Fig. 2 the results of Eq. 18 are reported for Z-Met enantiomers on the two columns. From these plots the same conclusions as for the full van Deemter curves can be made. Indeed SPPs show smaller contribution of $a(\nu )$+$c_{ads}\nu$ for the first enantiomer but larger contribution for the second enantiomer if compared to FPPs. Moreover, the latter curve is steeper for core-shell particles. This may be an indicator of a slower adsorption-desorption kinetics occurring with SPPs column. A plausible explanation could be found by taking into account some of the intrinsic differences of superficially porous particles compared to fully porous ones, reported in Table 1. From these data, it is clear that SP particles show 55% smaller specific surface area but 40% larger bonding density if compared to FP particles. The larger bonding density of chiral selector combined with the ability of Z-Met to fully fit and effectively enter the chiral pocket of teicoplanin could be the cause of a slower adsorption-desorption kinetics on the SPP-2.0 column, as it was recently demonstrated for other CSPs [6, 10, 34]. In terms of thermodynamic equilibria, this could suggest that the binding constant(s), i.e., the strength of the adsorption process between analyte and selector, could be larger on SPPs with respect to FPPs.

### Thermodynamic measurements

To further investigate the extent of adsorption-desorption kinetics on the two particle formats, adsorption isotherms have been calculated by means of IM on the two columns. Adsorption isotherms can provide pivotal information related to the heterogeneity of the stationary phase, the maximum amount of analyte that can be adsorbed and the intensity and strength of adsorption. This last thermodynamic point is related to kinetic characteristics of the CSP [31]. Indeed, it has been demonstrated that stronger bindings provoke on average longer adsorption-desorption times, which have a negative impact on the efficiency of the chromatographic separation, especially when high flow rates are employed [3, 6, 34].

**Fig. 3:**
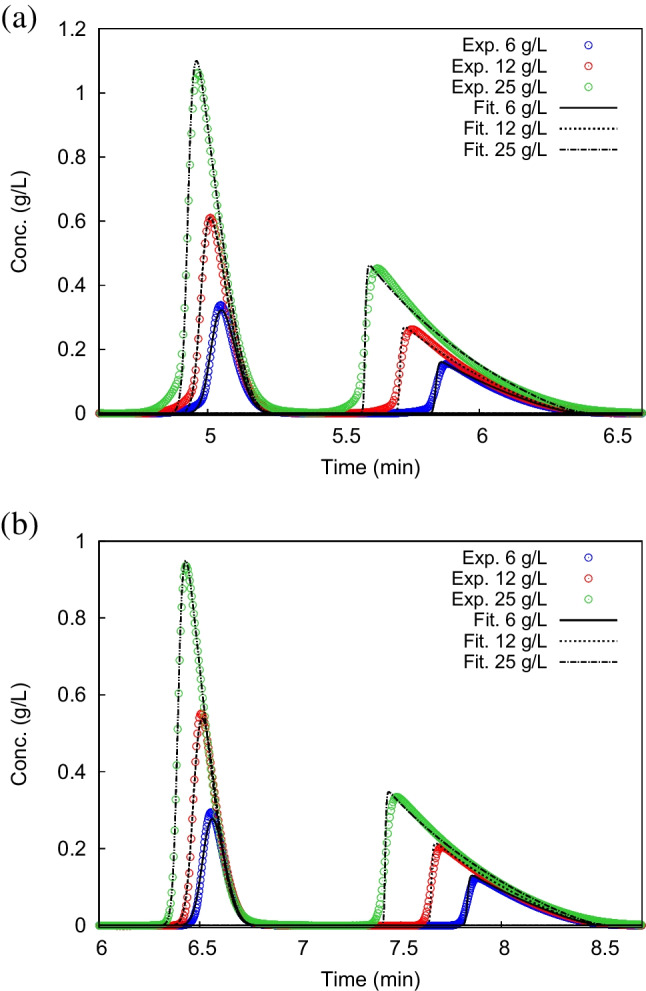
Experimental (empty circles) and calculated (lines) overloaded profiles of Z-Methionine enantiomers measured on SPP-2.0 (a) and FPP-1.9 (b) columns. Injection volumes: 5 $\mu$L. Concentrations: 6 g/L (blue), 12 g/L (red) and 25 g/L (green). Exp: experimental data, Fit: data calculated through Inverse Method

In this study, the competitive Bilangmuir isotherm model was found to be suitable for the description of the adsorption and separation of Z-Met enantiomers on the two columns employed in this study. Overloaded experimental (with points) peaks and profiles calculated through IM (with lines) are shown for SPP-2.0 (a) and FPP-1.9 (b) columns in Fig. 3. As it can be easily evinced, a very good agreement between experimental and theoretical peaks has been obtained. Calculated isotherm parameters are shown in Table 3. The first thing that can be noticed is that the SPP-2.0 column constantly shows smaller saturation capacities and stronger binding if compared to FPP-1.9 column. As a matter of fact, on the one hand, saturation capacities of the selective ($q^s_{sel}$) and nonselective ($q^s_{nsel}$) sites are more than 30% smaller for core-shell particles with respect to their counterpart. Since this parameter was calculated by taking into account only the porous zone (see the “Inverse method and Bilangmuir isotherm model” section), the values measured on the two columns were expected to be more or less the same. The discrepancy could be explained through the different pore accessibility due to the smaller particle porosity (-35%) and to the smaller surface area of SPPs (-56%). This provokes a reduced access of intraparticle volume for this type of particles which hinders the adsorption, leading to smaller $q^s$. On the other hand, binding constants on both selective and nonselective sites are always larger for SPP-2.0 column. These results are in accordance with [34], where it was found that a higher density of chiral selector was directly responsible for larger selective and nonselective bindings. This finding reveals important information related to adsorption-desorption kinetics, particularly relevant for ultra-fast applications of these CSPs.

**Fig. 4:**
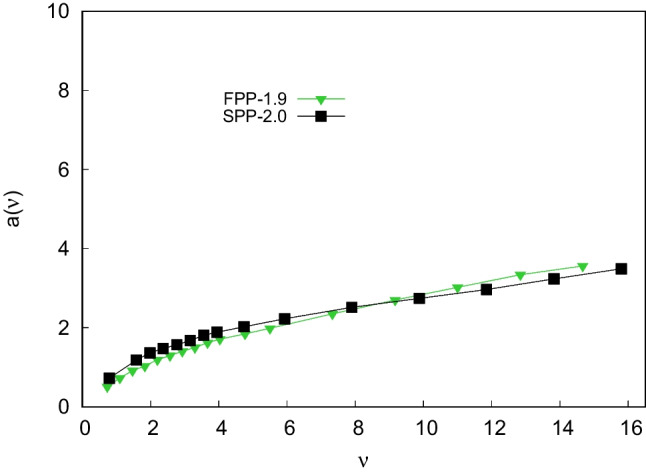
Average eddy dispersion contributions ($a(\nu )$) on FPP-1.9 column (green triangles) and SPP-2.0 column (black squares) as a function of the reduced interstitial velocity, $\nu$

By connecting the information concerning both the saturation capacities and the binding constants, it is possible to find out the extent of the different contributions to retention given by selective and nonselective sites. From data in Table 3 it can be evinced that the effect on the overall retention of first enantiomers given by selective sites, $a_{1,sel}=b_{1,sel}\,q^s_{sel}$, can be considered negligible, since $a_{1,sel} \ll a_{2,sel},a_{nsel}$, for both particle formats. This indicates that first Z-Met enantiomer does not experience selective sites and its retention is almost entirely given by other non-chiral (non-stereospecific) interactions with the CSP. This result confirms that a fast adsorption-desorption kinetics ($c_{ads}\rightarrow 0$) can be assumed for the first eluting enantiomer on both particle geometries.

### Adsorption-desorption kinetics

Based on the obtained results and data from literature, further considerations about the extent of adsorption-desorption kinetics can be made. Indeed, on the one hand, in the “Thermodynamic measurements” section, it has been demonstrated that firstly eluted enantiomers have a fast adsorption-desorption kinetics, since their interaction with selective sites can be considered negligible. Hence, Eq. 18 becomes:

19 $$\begin{aligned} a_1(\nu )=h_1+\frac{b}{\nu }+c_{s,1}\nu \end{aligned}$$

On the other hand, in Ref. [7], excess adsorption isotherms, describing the amount of a component of the binary mobile phase adsorbed at the solid-liquid interface in excess with respect to the amount in the bulk mobile phase, have been determined for the SPP-2.0 column using ACN/H_2_O mixtures as mobile phase. Results indicate that, at the mobile phase composition used in this study (85:15% ACN/H_2_O), water is preferentially adsorbed on the surface of the particle. Since this phenomenon depends on the functionalization of the particle and on the mobile phase components, the same considerations can be made for the FPP-1.9 column. Following these results, according to [38, 44] it has been experimentally proven that, when a water-rich layer is present on the surface of the particle, analyte mobility (and diffusion) is reduced due to the high viscosity of water compared to that of the bulk phase. As a consequence, the change of intraparticle diffusivity with the retention factor is very small, hence eddy dispersion is only barely influenced by analyte retention, unlike what happens with reversed phase (RP) chromatography. Hence, under these chromatographic conditions and with this specific system of analyte-selector, it is reasonable to assume that the contribution of eddy dispersion is independent from the analyte, i.e., it is the same for each enantiomer. As a consequence, the slope of the curve obtained through the subtraction of Eq. 18 for the second enantiomer and Eq. 19 for the first enantiomer (i.e., $a_2(\nu )+c_{ads}-a_1(\nu )$) will lead to the quantification of the $c_{ads}$ term (see Table 2) for both columns. From these data it can be evinced that the contribution of $c_{ads}$ for the SPP-2.0 column is double that of the FPP-1.9 column, as it was previously hypothesized, with a detrimental effect on the efficiency of the second eluted enantiomer. This indicates that the adsorption-desorption process on SP particles takes a significant amount of time with respect to FP particles. Consequently, the adsorption rate constant, $k_a$, is smaller for core-shell particles (see Eq. 9). This finding can be probably attributable to the higher selector loading observed for SPPs, that causes stronger bindings and slower adsorption-desorption processes for the enantiomer that perfectly fits the chiral pocket of the selector.

Once the $c_{ads}$ terms have been calculated, the sole contribution of eddy dispersion for the second eluted enantiomer, $a_2(\nu )$, can be obtained. In Fig. 4 eddy dispersion curves, calculated as the mean value between $a_1(\nu )$ and $a_2(\nu )$, for the two columns are illustrated. From this plot it can be easily observed that both particle formats show similar and very small eddy dispersion contributions, as expected.

## Conclusions

The importance of this work lies in the fact that it shows that the adsorption-desorption kinetics of selectand-selector systems in chiral chromatography, on a zwitterionic teicoplanin CSP, depends on the specific bonding density of chiral selectors. The specific bonding density of chiral selectors in turn has been found to depend on the geometry of particles employed as base materials for the preparation of the CSP. By a combination of thermodynamic and stop-flow measurements, the contribution to band broadening due to the adsorption-desorption kinetics has been quantified by means of a direct approach not involving specific theoretical assumptions. Thus, the information was gathered exclusively through chromatographic means under relevant experimental conditions that are the same in which the CSP is employed. Incidentally, this is almost never the case when kinetic data are obtained by spectroscopic measurements.

Even though the understanding of the adsorption-desorption kinetics in chiral chromatography does require additional studies involving different analyte-selector systems and experimental conditions, these results point out that the specific loading of chiral selectors not only affects the enantioselectivity (as expected), but it also contributes to the overall kinetic performance of the CSP. Therefore, the design and preparation of next-generation CSPs suitable for ultra-high efficient, ultra-fast separations cannot be separated from the deep investigation of very challenging topics, such as the preparation of CSPs with a fine control on the bonding density of chiral selector; the relationship between particle geometry and the extent of functionalization and the investigation of the radial homogeneity of functionalized particles in terms of chiral selector distribution; the relationship between pore size/degree of particle functionalization/accessibility of pores by molecules.
